# Supplementary material for: Restriction-Assembly: A Solution to Construct Novel Adenovirus Vector
Source: Viruses. 2022 Mar 6;14(3):546. doi: 10.3390/v14030546 (PMC8954691; doi:10.3390/v14030546)
Supplement: Supplementary file 1 [file viruses-14-00546-s001.zip › viruses-1588805-supplementary/viruses-1588805-supplementary220304.pdf]

A

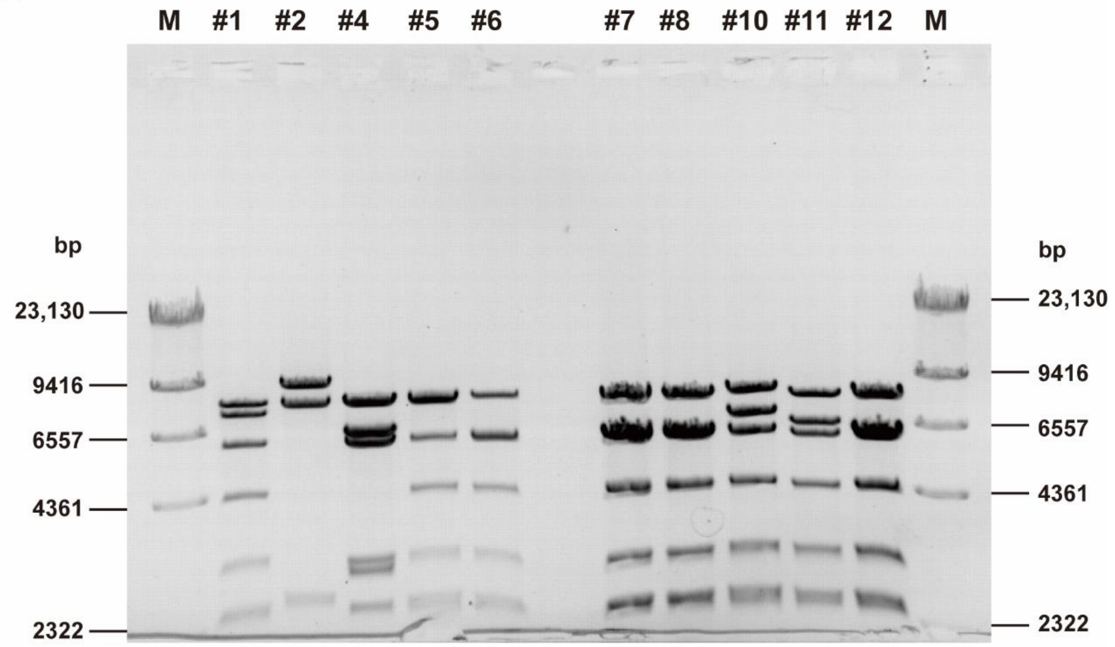

B

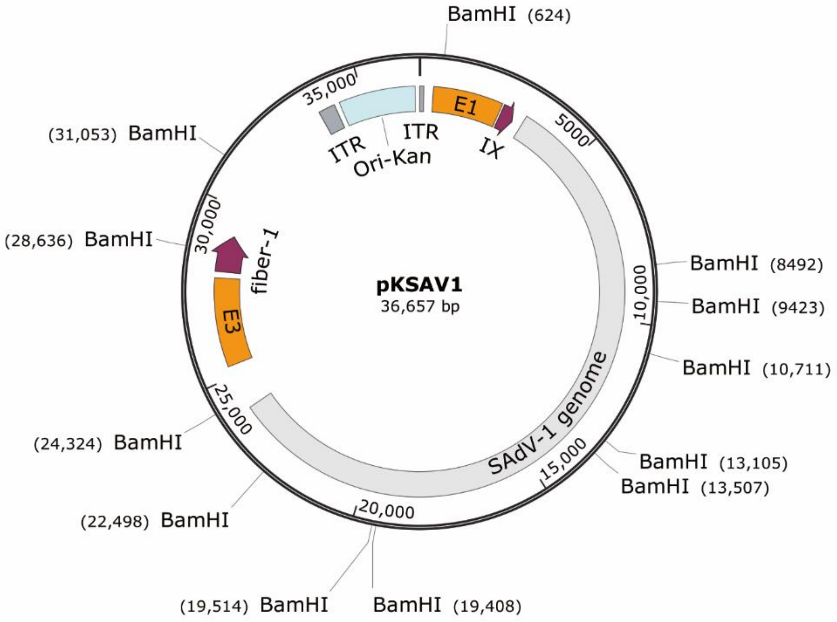

Figure S1. Restriction analysis of simian adenovirus 1 (SAdV-1) infectious clones. Plasmid backbone was amplified by PCR, mixed with SAdV-1 genomic DNA and subjected into Gibson assembly to generate infectious clones. (**A**) The plasmids (pKSAV1#1-#12) were extracted from 12 bacterial colonies, plasmids with high molecular weight were digested with BamHI and resolved on 0.7% agarose gel by electrophoresis. The predicted molecular weights (bp) of the bands were 106, 402, 931, 1288, 1826, 2394, 2417, 2984, 4312, 5901, 6228 and 7868. (**B**) The map of pKSAV1 with BamHI sites shown.

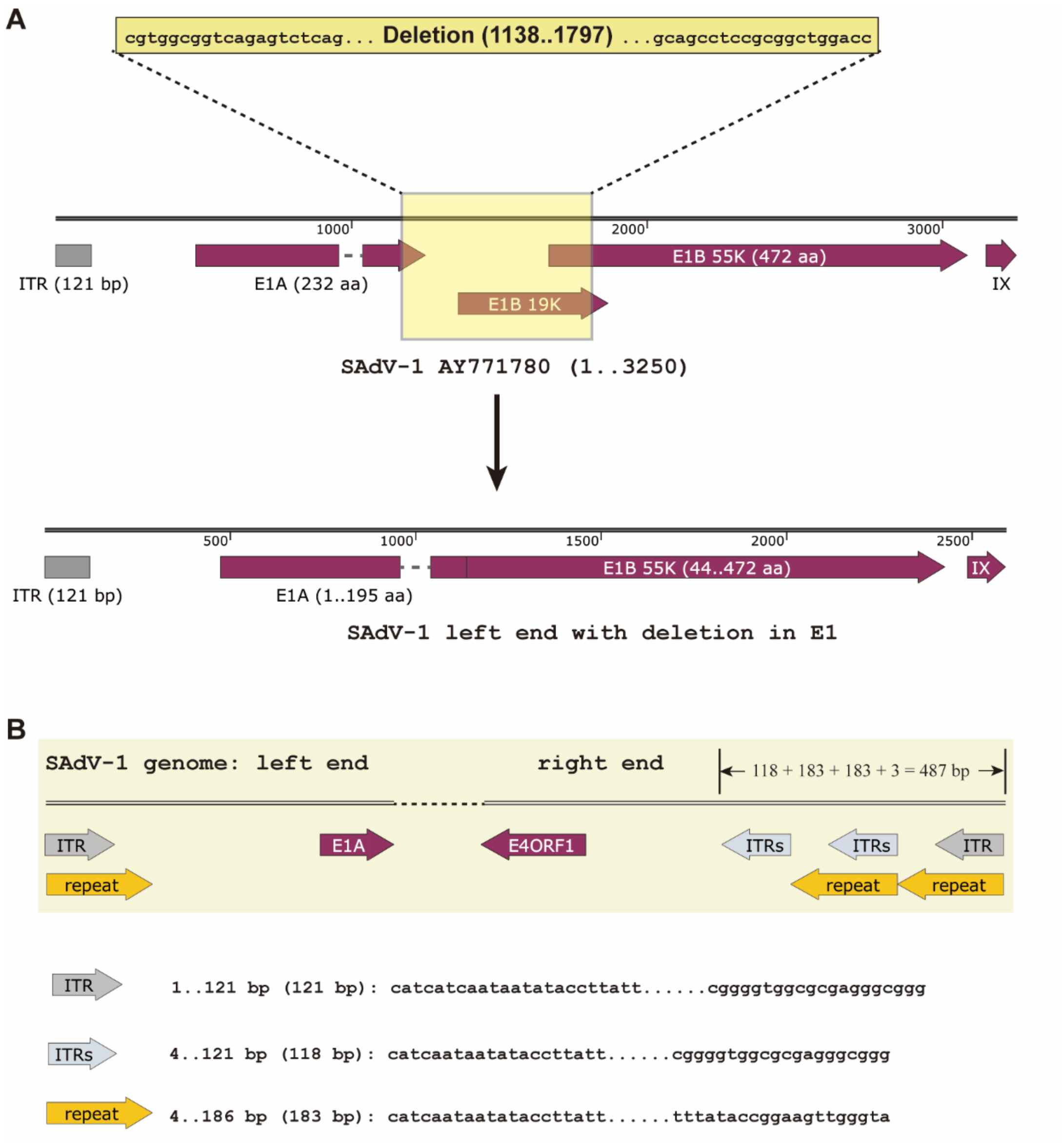

Figure S2. The mutations of SAdV-1 genome in infectious clone pKSAV1 when compared to the reported one in GenBank (accession no. AY771780). (**A**) a deletion of 660 bp in the E1 region. (**B**) repeat sequences found in the region of right ITR (inverted terminal repeat).

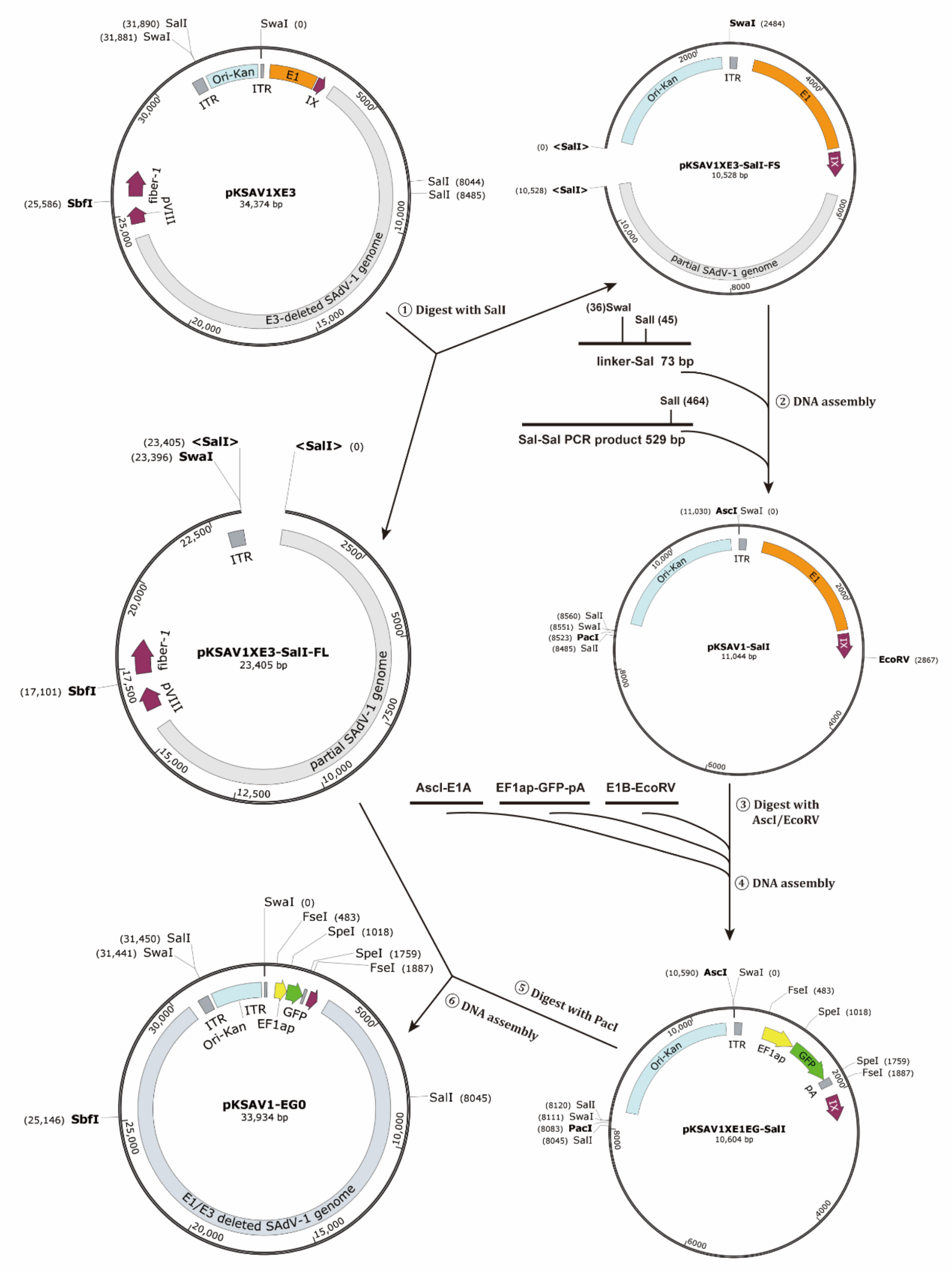

Figure S3. Schematic diagram of constructing adenoviral plasmid pKSAV1-EG0.

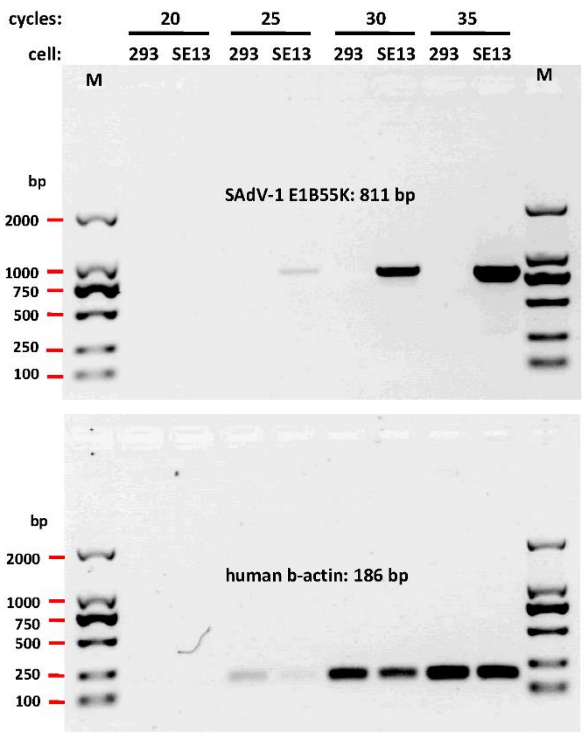

Figure S4. Detect the transcription of SAdV-1 E1B55K in 293SE13 cells by semi-quantity PCR. 293 or 293SE13 (SE13) cells were lysed in TRIzol reagent (Thermo Fisher, Cat. 15596026). The RNA was extracted, treated with DNaseI (Thermo Fisher, Cat. 18068-015), reversed transcribed to cDNA with oligo(dT)18 as the primer (PrimeScript II 1st Strand cDNA Synthesis Kit, Cat. 6210A; Takara), 1:40 diluted in water and used as the PCR templates. Semi-quantitative PCR was employed to detect the transcription of SAdV-1 E1B55K with primers of 2101SAV1E1Bs3 and 2101SAV1E1Bs4 (Table 1), and the transcription of human b-actin gene (GenBank NM_001101) was also determined and served as a template loading control [51]. The primers for human b-actin were 1304actin-F (tggcacccagcacaatgaa) and 1304actin-R (ctaagtcatagtccgcctagaagca). M: DL2000 DNA marker (Takara).

Reference:

51 Kinoshita, T., Imamura, J., Nagai, H., Shimotohno, K. Quantification of gene expression over a wide range by the polymerase chain reaction. *Anal. Biochem*. **1992**, *206*, 231–235. https://doi.org/10.1016/0003-2697(92)90358-E

.

Table S1. Summary data of plasmid construction. The restriction-assembly procedure of plasmid construction included fragment preparation, DNA assembly reaction, bacterial transformation, plasmid extraction, restriction analysis and sequencing confirmation. The large DNA fragment for DNA assembly exclusively originated from restriction enzyme-digested (RD) parental plasmid, while the small fragment could be from RD or PCR products. Generally, 20-100 colonies could grow on LB plate after transforming E. coli competent cells with DNA assembly product; E. coli colonies were randomly picked up and propagated in liquid antibiotic-containing LB media, and plasmid DNA was extracted and resolved on agarose gel; the plasmids with expected size were further digested with restriction enzymes and the bands pattern of digested plasmid was photographed and analyzed after electrophoresis; and finally, the whole plasmid or PCR products covering the fusion sites or PCR-amplified regions in the plasmid were subjected into Sanger sequencing.

**Product plasmid**	**size (bp)**	**DNA assembly reaction**		**plasmid extraction**	**restriction analysis**	**Sanger sequencing**
		**large fragment (bp)**	**small fragment (bp)**	**(colonies picked/with expected size)**	**(digested/correct)**	**(sequenced/correct)**
pKSAV1	36,657	34,450	2563 (PCR)	18/15	15/15	1/1
pKSAV1-AscI	13,724	13,656	117 (PCR)	4/4	4/4	-
pKSAV1XE3CGA-AscI	12,853	7346	2680; 1442; 1522 (PCR)	8/8	8/8	1/1
pKSAV1XE3CGA	35,786	23,001	12,853 (RD)	4/4	4/4	-
pKSAV1ME1-SalI	11,044	10,528	529; 73 (PCR)	8/8	5/5	-
pKSAV1XE1EG-SalI	10,604	8163	1029; 1528 (PCR)	8/8	8/8	-
pKSAV1EG0	33,934	23,405	10,604 (RD)	8/8	8/8	1/1
pKSAV1EG	33,568	31,333	997; 1324 (PCR)	6/6	6/6	1/1
pcDNA3TSAV1-E1B55K	6992	5574	1466 (PCR)	6/5	6/5	3/3
